# Elder Justice and Nursing Home Reforms 2020

**DOI:** 10.1093/geroni/igaa057.2549

**Published:** 2020-12-16

**Authors:** Lori Smetanka

**Affiliations:** The National Consumer Voice for Quality Long-Term Care, Washington, District of Columbia, United States

## Abstract

The presentation will provide perspective on the current state of care quality in long-term care facilities and the legislative discussions and actions of the 116th Congress.

